# Feather, But Not Plasma, Glucocorticoid Response to Artificial Light at Night Differs between Urban and Forest Blue Tit Nestlings

**DOI:** 10.1093/icb/icab067

**Published:** 2021-07-17

**Authors:** Davide M Dominoni, Dylon Teo, Claire J Branston, Aryan Jakhar, Bedur Faleh A Albalawi, Neil P Evans

**Affiliations:** 1 Institute of Biodiversity, Animal Health and Comparative Medicine, University of Glasgow, Glasgow G128QQ, UK; 2 Indian Institute of Science Education and Research, Thiruvananthapuram, Kerala 695551, India; 3 Department of Biology, University of Tabuk, Tabuk 47512, Saudi Arabia

## Abstract

Urbanization drives phenotypic variation in many animal species. This includes behavioral and physiological traits such as activity patterns, aggression, and hormone levels. A current challenge of urban evolutionary ecology is to understand the environmental drivers of phenotypic variation in cities. Moreover, do individuals develop tolerance to urban environmental factors, which underlie adaptative responses and contribute to the evolution of urban populations? Most available evidence comes from correlative studies and rare experiments where a single urban-related environmental factor has been manipulated in the field. Here we present the results of an experiment in which we tested for differences in the glucocorticoid (CORT) response of urban and rural blue tits nestlings (*Cyanistes caeruleus*) to artificial light at night (ALAN). ALAN has been suggested to alter CORT response in several animal species, but to date no study has investigated whether this effect of ALAN differs between urban and rural populations. Immediately after hatching, urban and forest broods were either exposed to 2 lux of ALAN (using an LED source mounted inside the nestbox) or received no treatment (dark control). The experiment lasted until the chicks fledged. When the chicks were 13 days old plasma samples were collected to measure baseline CORT concentrations, and feather samples to provide an integrative measure of CORT during growth. Forest birds had higher plasma CORT (pCORT) concentrations than their urban counterparts, irrespective of whether they were exposed to ALAN or not. Conversely, we found population-specific responses of feather CORT to ALAN. Specifically, urban birds that received ALAN had increased feather CORT compared with the urban dark controls, while the opposite was true for the forest birds. pCORT concentrations were negatively associated to fledging success, irrespective of population and treatment, while feather CORT was positively associated to fledging success in broods exposed to ALAN, but negatively in the dark control ones. Our results demonstrate that ALAN can play a role in determination of the glucocorticoid phenotype of wild animals, and may thus contribute to phenotypic differences between urban and rural animals.

## Introduction

Urbanization represents a major modification of natural habitats and is considered as a threat to biodiversity (Grimm et al. 2008). While some wild species that possess specific traits are able to colonize and even thrive in urban areas, others are driven away when facing urban sprawl (McKinney 2006). Within the species that do well in the urban environment, some readily exploit anthropogenic resources, such as food supplementation and artificial nesting sites, whereas others suffer from pressures imposed by city life, although they might appear to breed successfully. Understanding the effects of such pressures, such as noise, artificial light at night (ALAN), impervious surface, air pollution on life-history traits, behavior, physiology, and population dynamics, has been a major focus of urban ecology (Aronson et al. 2014; Alberti et al. 2017; Johnson and Munshi-South 2017; Ouyang et al. 2018b).

The magnitude and direction of the effects of urbanization have been shown to vary depending on both intrinsic (e.g., species, sex, age, and body condition) and extrinsic factors (e.g., city location and age, human population density, and amount of a particular urban environmental factor) (Beninde et al. 2015). A clear example for this large variation in urban effects is the relationship between urbanization and physiological stress, as measured by baseline or stress-induced glucocorticoid concentrations. Glucocorticoids are secreted by the adrenal gland after activation of the hypothalamic–pituitary–adrenal (HPA) axis and are considered to represent the allostatic demand on an animal (Romero 2004; Crespi et al. 2013; MacDougall-Shackleton et al. 2019). Thereby, glucocorticoids have been widely used in urban ecological studies that have investigated effects of the urban environment on wildlife health and fitness (Bonier 2012; Iglesias-Carrasco et al. 2020). Urban life is often regarded as “stressful” for wild species, but an emerging body of evidence suggests that this may not always be true. Several reviews and meta-analyses have highlighted the lack of a clear pattern between urbanization and markers of physiological stress, with urban populations of wild species having higher, lower, or equal glucocorticoid concentrations when compared with rural conspecifics (Bonier 2012; Ouyang et al. 2018b; Murray et al. 2019; Iglesias-Carrasco et al. 2020; Injaian et al. 2020). For instance, a meta-analysis by Murray et al. (2019) demonstrated a negative effect of urbanization on toxicant load and parasitism, but glucocorticoid concentrations were unaffected. Much of the previous work in this area has been correlational and has focused on the comparison of physiological stress between limited numbers (usually only two) urban and rural populations of the same species. To discern the causes and consequences of variation in glucocorticoid concentrations between urban and rural populations, more experimental work is required, in which urban-specific environmental factors are manipulated in a controlled manner and the impact on glucocorticoid concentrations are determined.

Among environmental factors typical of cities, ALAN has attracted a lot of recent attention. Light is a fundamental source of both energy and temporal information, and as such many biological processes rely on it (Gaston et al. 2013, 2017). Within the urban environment, light patterns are fundamentally different relative to the natural environment and the dramatic increase in artificial lighting observed over the last century (Kyba et al. 2017) has been shown to impact behavioral, physiological, and molecular functions in several species (Rich and Longcore 2006; Dominoni et al. 2020a; Falcón et al. 2020; Sanders et al. 2021). The effect of ALAN on endocrine function has generated significant interest. Several studies have characterized the impact of ALAN on glucocorticoid concentrations (Ouyang et al. 2018a). A recent cross species meta-analysis concluded no overall effect of ALAN on stress responses (Sanders et al. 2021). However, this analysis did not rule out the possibility that specific taxa might be more sensitive to the effects of ALAN than others. Indeed, all avian studies published to date have reported an increase of baseline corticosterone (CORT) concentrations in response to ALAN (Ouyang et al. 2015; Russ et al. 2015; Alaasam et al. 2018; Mishra et al. 2019; Grunst et al. 2020; Malek et al. 2020). It is of note that the effects of ALAN in birds are wavelength-specific, as effects were only seen with short wavelength light with amber and red light having no effect on CORT concentrations (Ouyang et al. 2015; Alaasam et al. 2018). The majority of the studies of the effects of ALAN on physiological stress has either been conducted in captivity (Alaasam et al. 2018; Mishra et al. 2019; Malek et al. 2020), or in unlit areas where birds had likely not previously been exposed to ALAN (Ouyang et al. 2015; Grunst et al. 2020). A single study has investigated the effects of ALAN along a light pollution gradient (Russ et al. 2015). Although the results of this study indicated that CORT increased with ALAN, effects of other environmental factors that co-vary with urbanization could not be excluded (Russ et al. 2015).

When assessing effects of the urban environment, such as ALAN on stress and fitness, an additional factor that must be considered is the possibility that urban populations could develop tolerance (i.e., decreased sensitivity) to ALAN over time, as a result of adaptive acclimation (non-genetic change) or micro-evolution. Such changes could result in equal or even lower glucocorticoid secretion (baseline and in response to challenges) compared with non-urban conspecifics. Indeed, population-specific responses to urbanization and urban environmental factors by the HPA axis have been documented previously. For instance, two different common-garden experiments have shown lower stress responses in urban, compared with non-urban, populations of European blackbirds (*Turdus merula*) (Partecke et al. 2006) and dark-eyed juncos (*Junco hyemalis*) (Atwell et al. 2012). Furthermore, a recent study on house wrens (*Troglodytes aedon*) showed that CORT concentrations increased after traffic noise exposure in rural but not urban populations (Davies et al. 2017), which would suggest that population-specific CORT responses can also be linked to urban-specific environmental factors. Whether stress responses to ALAN differ between urban and rural bird populations has not been tested.

This study investigated the impact of ALAN on CORT concentrations of urban and forest blue tit nestlings (*Cyanistes caeruleus*). Nestlings were exposed to ALAN from hatching until fledging, using nests located in multiple urban and forest sites in and around Glasgow, UK. Feather and plasma samples were collected when the chicks were 13 days old to measure CORT concentrations. Plasma CORT (pCORT) concentrations were used to provide a measure of allostatic load on the day of sample collection (Fairhurst et al. 2013; Romero and Fairhurst 2016). The CORT concentration in feathers (fCORT) provides an integrated measure of the activity of the HPA axis as a physiological response to stressors experienced during the period of feather growth. Thus, it provides information on the total baseline and stress-induced CORT secreted during this time, and provides a measure of the HPA responses to past environmental conditions, from the start of feather growth (soon after hatching) until the day of sample collection (Fairhurst et al. 2013; Legagneux et al. 2013; Jenni-Eiermann et al. 2015; Romero and Fairhurst 2016; Fischer et al. 2017). Using these samples, this study sought to address the following questions:

1. Do plasma and feather CORT concentrations differ between urban and forest blue tit nestlings?
2. Is the CORT response of nestlings to ALAN population-specific?
3. Do CORT concentrations with and without ALAN predict fledging success?

We hypothesized that plasma and feather CORT concentrations prior to and up to fledging would not differ between urban and forest nestlings, but that forest nestlings would show increased CORT concentrations in response to ALAN compared with dark control nestlings, while urban nestlings would not. We predicted that these effects should be stronger for fCORT compared with pCORT, as fCORT would represent an integrated measurement of CORT levels over the duration of feather growth. Finally, we hypothesized that higher CORT concentrations in both feather and plasma samples would be related to lower fledging success, as shown in several previous studies conducted with tit species (Ouyang et al. 2011, 2015; Davies et al. 2017).

## Materials and methods

### Ethical statement

The experiment was conducted under the UK Home Office regulation (project license 70/7899), under the authority of Scottish Natural Heritage (permit number 52463) and the British Trust for Ornithology (ringing licenses to D.M.D. and C.J.B.).

### Study sites and bird field work

The study took place at two forest and four urban sites in and around Glasgow, UK (see Supplementary Table S1 and Supplementary Fig. S1 for details of sites) in April–June 2019. Hereafter, habitat refers to urban or forest environments, while site refers to the specific area where a nest was located. Forest sites were located within the Loch Lomond and The Trossachs National Park, and were all oak-dominated woodlands, albeit with different understory (Supplementary Table S1). Urban sites were located within the Glasgow City and East Dunbartonshire council areas, but differed in their amount of impervious surface, green cover, and distance to the city center (Supplementary Table S1).

At each site, existing nestboxes (Woodcrete Schwegler boxes, hole size = 32 mm) were used (Supplementary Table S1). Nestboxes were located approximately 25 m from each other and at approximately 2 m height. Nestboxes were monitored weekly during the entire reproductive period, and all reproductive activities were recorded. Close to the expected hatching date, nests were monitored every day to identify the exact hatching date. After hatching and light treatment (see below), broods were left undisturbed until day 13, when chicks were weighed, ringed, and blood and feather samples obtained (see below). Twenty days after hatching, or later if broods had not fledged, the boxes were cleaned and checked for any remaining dead nestlings. Based on these data, fledging success was defined as the number of chicks counted on post-hatching day 13, minus any dead young found during clean-out, divided by number of hatchlings. All broods in this experiment were first broods (blue tits rarely have second broods in Scotland). Blue tit chicks typically fledge at 18 days of age and, in our populations, lay an average of nine eggs. Both clutch size and fledging success are usually higher in the forest than in the city sites (Pollock et al. 2017; Jarrett et al. 2020). Nestbox temperature does not differ between our urban and forest sites (Dominoni et al., in review).

### Light treatment

As soon as the first chick hatched, the whole brood was either exposed to ALAN (experimental group) or left unexposed (dark control group). Broods within the same habitat were assigned to the experimental or dark control group in alternate order. The forest and urban populations studied do not differ in egg-laying date (Capilla-Lasheras et al. 2017; Pollock et al. 2017) and thus there was no temporal biases between either treatments or habitats. All broods were transferred, in their original nest cups, into new nestboxes that were equipped with a single cool-white LED bulb mounted on the ceiling, powered by a 12 V, 3.2 Ah battery placed into a plastic bag to prevent water ingress and located outside of the nestbox. The light was left off in the dark control nestboxes. For experimental nestboxes, all lights were checked, and standardized to 2 lux before deployment. Because of technical limitations, the lights could not be turned off during the day, which we recognize could also have an impact on daytime behavior and physiology. However, we also note that, depending on nestbox orientation, daytime light intensity immediately inside the entrance hole can reach up to 10 lux. The light was on until the chicks fledged. The photoperiod at the average time of hatching (May 11 for both populations) was 16 h and 3 min. We used 18 control nestboxes (nine in the forest and nine in the city) and 17 ALAN nestboxes (nine in the forest and eight in the city).

### Blood and feather sampling

Blood and body feathers were obtained during the morning (8:00–12:00) 13 days after first hatching. For every nest, three random nestlings were removed from the brood and a blood sample collected from each individual within 3 min of opening the nestbox. The blood sample was collected by puncture of the wing vein with a sterile needle and collection of blood into a _**∼**_75_** µ**_L capillary. The blood sample was subsequently emptied into a 1.5 mL Eppendorf tube and maintained on wet ice until returned to the laboratory, where samples were spun for 10 min at 10,000 rpm to separate plasma from red blood cells. Plasma was then transferred into a new 1.5 mL tube and placed in a −80_**°**_C freezer until analysis. After collection of the blood sample, five feathers were collected from the belly of each nestling and placed into a 1.5 mL Eppendorf tube. Feather samples were collected from all remaining nestlings in each brood, thus sample numbers for feathers exceed those for plasma. Feather samples were stored in the dark until analysis. Nestlings did not differ in body mass between habitats (linear mixed model [LMM], *F*_1,31_ = 1.99, *P* = 0.17) or treatment groups (*F*_1,30_ = 0.33, *P* = 0.57). The sampled nestlings did not differ in body mass from the nestlings that were not sampled in the same brood **(**LMM, *F*_1,30_ = 0.20, *P* = 0.65).

### Feather CORT measurements

Feather samples were washed with 1 mL of 20% methanol and then twice with UltraPure water each for 10 min in an orbital shaker before being left to air-dry. Washing rids the feathers of dirt, contamination, and CORT from other sources such as feces or preening oils. Once dried, the calamus of the feathers was removed and the feathers cut in to less than 5 mm lengths and weighed (Mettler AE160). CORT was extracted from the feathers using a modification of a previously described method (Bortolotti et al. 2008). Briefly, 3 mL of high performance liquid chromatography (HPLC)-grade methanol was added to each feather and it was incubated at 52_**°**_C in an orbital shaker at 175 rpm for 19 h. After incubation, 1 mL of methanol was removed into a 12 × 75 mm borosilicate glass tube and dried in a sample concentrator (Savant SC210A SpeedVac Concentrator). The sample was reconstituted (multi-vortex for 10 min) in 150_** µ**_L of assay buffer. The CORT concentration was then measured for each bird sample (thus pooling all feathers for a bird into the same sample) using a commercial ELISA kit (Cayman Chemical Company, CORT ELISA kit, Item No. 501320), following the manufacturer’s instructions. Mean assay sensitivity (nine assays) was 3.02 pg/mg and inter- and intra-assay coefficients of variation averaged 8.20% and 7.23%, respectively. Mean feather weight was 0.23 mg (sd = 0.99, min = 0.4, max = 5, Supplementary Fig. S2). Feather weight was not significantly related to fCORT concentration (*P* = 0.91, Supplementary Fig. S2). This assay was previously validated in our laboratory (Albalawi 2020) and data showing parallelism within the assay are provided in the Supplementary Materials (Supplementary Fig. S3).

### pCORT measurements

CORT was extracted from plasma following a standard diethyl ether extraction. Ten microliters of plasma and 40_** µ**_L of assay buffer were vortexed with 1 mL of diethyl ether. The solvent was decanted using a methanol-dry ice bath and dried down in a sample concentrator before reconstitution in 150_** µ**_L of assay buffer, as above. CORT concentrations were measured using a commercial ELISA kit (Cayman Chemical Company, CORT ELISA Kit, Item No. 501320), following the manufacturer’s instructions. Mean assay sensitivity (four assays) was 1.63 pg/mL and inter- and intra-assay coefficients of variation averaged 8.95% and 9.91%, respectively.

### Statistical analyses

The analyses were conducted in R version 3.6.3 (R Development Core Team 2015) using LMMs or generalized linear models (GLMs) depending on the response variable, with the lme4 package (Bates et al. 2015). A backward selection process was used, starting off with global models that contained all biologically meaningful fixed and random effects, as well as interactions between fixed effects. The significance of fixed effects and interactions was tested by comparison of models with and without a term of interest using likelihood ratio tests implemented in the function *step* and *drop1* in R. Model assumptions were confirmed by visual inspection of the QQ plot of the residuals from the final model as well as by plotting residuals over fitted values to check for heteroskedasticity. Whenever a significant categorical fixed effect (main term or in interaction with another term) was found, *post hoc* pairwise comparisons were performed using the function *emmeans* in the package emmeans (https://cran.r-project.org/web/packages/emmeans/index.html). Two groups were assumed to be significantly different from each other if the estimated mean of one group was not included in the 95% confidence intervals (CIs) of the other.

Variation in pCORT and fCORT concentrations was tested using LMMs. CORT values were log-transformed. Habitat, treatment (and their interaction), nestling body mass, hatch date of the brood, and brood size were included as fixed effects. Nestbox and site were included as a random effect to account for non-independency of data collected on nestlings from the same brood and site.

The effect(s) of pCORT and fCORT on reproductive fitness levels were tested using two separate GLMs. Because of the strong differences in clutch size and number of fledglings raised in the urban and forest broods at the study sites (Pollock et al. 2017), fledging success (number fledglings/number of hatchlings) was thought to represent a more biologically meaningful variable to explore the effects of the nestling treatment (after hatching) and of CORT concentrations on reproductive success. CORT values were log-transformed and included as fixed effect. Additional fixed effects included were habitat, treatment, hatch date of the brood, brood size, and the two-way interactions between CORT concentrations and treatment as well as habitat.

Last, relationship between pCORT and fCORT concentrations was tested using a Pearson correlation test.

## Results

**Fig. 1.**
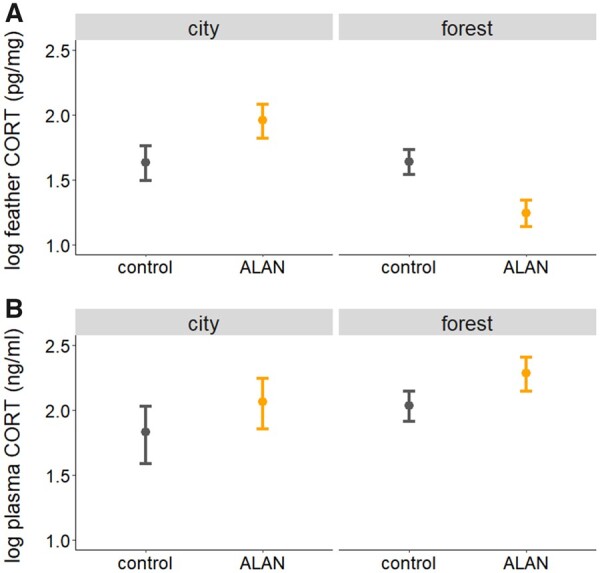
The effect of ALAN and habitat on CORT concentrations. (**A**) ALAN affected fCORT concentrations in a habitat-dependent manner. fCORT concentrations were higher in city birds exposed to ALAN, while they were lower in forest birds exposed to ALAN (*N* = 139 nestlings. Control: forest = 34, city = 30; ALAN: forest = 43, city = 32). (**B**) pCORT concentrations were affected by habitat, with forest birds having higher concentrations than city birds, but they were unaffected by light treatment (*N* = 99 nestlings. Control: forest = 23, city = 26; ALAN: forest = 27, city = 23). CORT concentrations were log-transformed to reach normality of residuals. Points and error bars represent means ± SEM. Asterisks denote the *P*-values of significant differences between pairwise combinations (***<0.001, **0.001–0.01, and *0.01–0.05).

fCORT concentrations were significantly affected by the interaction of treatment and habitat *(***_*β*_ **= 0.6, *t* = 2.1, *P* = 0.044, ***Fig. 1***A and Supplementary Table S2). *Post hoc* tests indicated that fCORT concentrations in forest populations were significantly higher in dark control compared with ALAN nestlings (dark control mean, lower–upper CIs: 1.49, 1.19–1.79; ALAN mean, lower–upper CIs: 1.18, 0.89–1.47; Supplementary Table S3). Conversely, in the city, dark control birds had significantly lower concentrations of fCORT than ALAN birds (dark control mean, lower–upper CIs: 1.51, 1.20–1.82; ALAN mean, lower–upper CIs: 1.82, 1.51–2.14; Supplementary Table S3). ALAN nestlings in the city had significantly higher fCORT concentrations compared with the ALAN nestlings in the forest, while fCORT concentrations in the dark control birds did not differ between the two populations (Supplementary Table S3).pCORT concentrations were significantly higher in the forest than in the city *(***_*β*_ **= 0.4, *t* = 2.6, *P* = 0.009, Fig. 1B and Supplementary Tables S4 and S5), were negatively associated with body mass (**_*β*_** = −0.23, *t* = −2.9, *P* = 0.004, Supplementary Table S4), but were not affected by the ALAN treatment (**_*β*_** = −0.1, *t* = −0.7, *P* = 0.473).

**Fig. 2.**
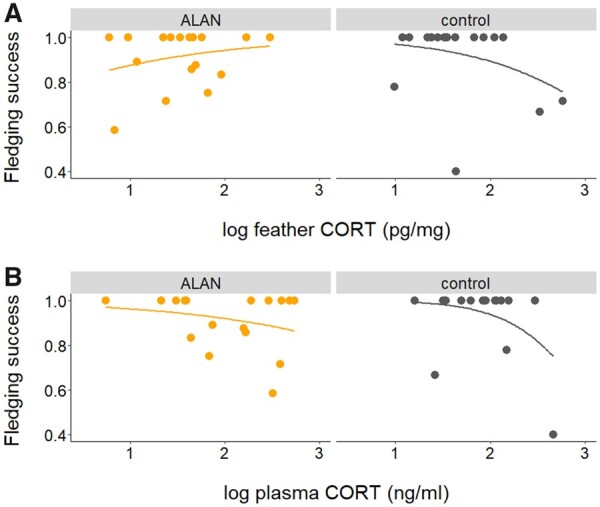
Relationships between CORT concentrations and reproductive success. (**A**) The interaction of fCORT with the light treatment had a significant effect of fledging success (number of fledglings/number of hatchlings). Increasing fCORT concentrations were positively related to fledging success in broods exposed to ALAN inside the nestbox, while the opposite was true for broods not exposed to ALAN (*N* = 35 nestboxes). (**B**) pCORT concentrations were negatively related to fledging success in both light treatment groups (*N* = 33 nestboxes). Both fCORT and pCORT concentrations were logtransformed in the original models.

Fledging success was significantly affected by the interaction of treatment and fCORT *(***_*β*_** = −4.4, *z* = −3.5, *P* < 0.001, ***Fig. 2***A and Supplementary Table S6). With increasing concentrations of fCORT, fledging success increased in the ALAN group but decreased in the dark control group (Fig. 2A). pCORT was negatively related to fledging success, but independently of treatment and habitat *(***_*β*_** = −1.5, *z* = −2.8, *P* = 0.005, Fig. 2B and Supplementary Table S7). In both of the models that related fledging success to CORT (either fCORT or pCORT), fledging success was affected by habitat, with forest parents raising more fledglings than city parents (see Supplementary Tables S6 and S7 for full results).

Finally, concentrations of fCORT and pCORT were not correlated (Pearson correlation coefficient = 0.01, *P* = 0.92, Supplementary Fig. S4).

## Discussion

The results of this study show that ALAN can have different effects on CORT concentrations of nestling blue tits in urban compared with rural environments. Higher pCORT concentrations on day 13 were correlated with a lower probability of fledging and a lower body mass, which is in agreement with the results of previous studies (Ouyang et al. 2013, 2015). However, in nestlings exposed to ALAN fCORT, which represents a measure of integrated HPA activity over the duration of feather growth, was positively associated with fledging success. This result suggests that while instantaneous CORT concentrations close to the time of fledging may relate to fledging success, ALAN has unexpectedly a positive effect upon putative fitness of wild blue tit nestlings.

### Plasma, but not feather, CORT concentrations are higher in the forest than in the city

The results of this study assessed effects of ALAN on two measures of physiological stress and contribute to an increasing body of evidence that the relationship between urbanization and CORT concentrations is complex and may vary depending on the species, city, age, sex, and time of the year (Bonier 2012; Iglesias-Carrasco et al. 2020). The results demonstrated that pCORT concentrations on day 13 of life were higher in forest than urban blue tit nestlings, but the concentrations of CORT in feathers were not significantly different between forest and urban populations (although the direction of the effect is the same). To our knowledge, this is the first time that instantaneous and integrated measures of CORT have been analyzed simultaneously in blue tit nestlings in relation to urbanization. Moreover, much of the work to date on urbanization and CORT concentrations of birds has focused on adult and juvenile life stages. Those studies that did measure CORT in nestlings show mixed effects of urbanization. For instance, while pCORT did not vary between suburban and rural American crow nestlings (Heiss et al. 2009), rural red-winged blackbird nestlings had higher fecal CORT than urban ones (Buxton et al. 2018).

One potential explanation for why pCORT concentrations might be lower in urban than forest nestlings is that chronic stress during development could have dampened baseline pCORT (Rich and Romero 2005; Cyr and Romero 2007). Urban nestlings in a variety of species are often in poorer body conditions compared with their forest conspecifics (Liker et al. 2008; Sumasgutner et al. 2014; Bailly et al. 2016; Biard et al. 2017; Pollock et al. 2017), which might suggest chronic stress in early life. Previous studies with these same populations of blue tits have also reported that the nestlings suffer from poor nutrition, and exhibit delayed development, smaller body size at fledging, and higher mortality (Capilla-Lasheras et al. 2017; Pollock et al. 2017; Jarrett et al. 2020). However, this scenario is unlikely because elevated pCORT concentrations in our study were associated with a reduced fledging success and with a reduced body mass. Thus, high pCORT concentrations seem to be associated with a poor fitness and a low nutritional status. Moreover, lower pCORT because of chronic nutritional status would have led to low fCORT concentrations, but there was no difference between urban and forest birds in fCORT.

An alternative explanation could be that more stress reactive individuals, with very high concentrations of CORT, might have died prior to day 13. Selective disappearance of urban nestlings in poor conditions and with short telomeres has been shown for a closely-related species, the great tit (*Parus major*) (Salmón et al. 2017), but is unknown in blue tits. Given the high nutritional stress and mortality of blue tit nestlings in the urban population we studied (Pollock et al. 2017), it is conceivable to hypothesize that the nestlings who died in the early stages of development had higher CORT concentrations than those who made it until the day 13 of sampling. Missing these high-CORT individuals from our dataset could have therefore led to overall lower pCORT concentrations in our urban sample. Obtaining samples earlier in the nestling period would enable this hypothesis to be tested.

### The effects of ALAN on integrated CORT concentrations are environment-dependent

The results of the study indicated that environment was the only variable that had a significant effect on pCORT, the instantaneous measure of HPA activity. However, ALAN had environment-dependent effects on fCORT, which provides a measure of integrated CORT concentrations over the period of feather growth. Urban birds showed increased fCORT in response to ALAN compared with dark control birds, while the opposite was true for forest birds. These results are similar to those recently observed in a suburban population of great tits, where fCORT increased in nestlings exposed to ALAN (Grunst et al. 2020). These results suggest that urban blue tit nestlings did not hatch with a lower sensitivity to ALAN, contrary to what we hypothesized. One possibility, to explain the observed result, is that the higher HPA activity in the ALAN urban birds is adaptive. While there was no significant effect of the interaction between fCORT, habitat, and treatment on fledging success, the results did indicate a positive relationship between fledging success and fCORT in the ALAN birds, independently of the rearing habitat. The low sample size in this experiment may have prevented detection of a significant three-way interaction, and therefore this hypothesis warrants future investigations.

An additional explanation for the higher fCORT levels in response to ALAN in urban but not forest chicks is potential interactions between stressors, for example increased activity as a direct effect of ALAN and food limitation as an indirect effect of the urban environment (Jarrett et al. 2020). Nutritional stress is known to elevate CORT levels of birds, but often this relationship is revealed only when other stressors are present (Lynn et al. 2003; Angelier et al. 2007). The combined effect of ALAN and poor diet in early life might have resulted in higher fCORT in the urban but not the forest population. In this case, lower fledging success might be expected in the urban broods, but the results of this study showed opposite patterns. While more experimental work is needed to investigate potential combined effects of ALAN and other urban variables (Dominoni et al 2020b; Senzaki et al. 2020), including diet, at present this hypothesis seems unlikely.

ALAN is known to decrease melatonin secretion and increase activity at night in diurnal birds (Dominoni et al. 2013b; de Jong et al. 2016) as well as many other diurnal species (Grubisic et al. 2019). In turn, increased activity is often linked to increase CORT secretion (Zani de Souza et al. 2001; Jessop et al. 2002; Tarlow et al. 2003). This increase in CORT may reflect the fact that one of the primary functions of CORT is energy mobilization, including regulation of carbohydrate metabolism (MacDougall-Shackleton et al. 2019). The higher fCORT observed in the urban nestlings in response to ALAN in this study might, therefore, be the result of increased nocturnal activity due to the presence of light during the night, as shown for great tits (Raap et al. 2016; Ouyang et al. 2017; Ulgezen et al. 2019; Grunst et al. 2020). Unfortunately, complementary activity data were not collected during this study to test this hypothesis, but we suggest that future studies could attempt to directly link increased nocturnal activity due to ALAN, reduced melatonin secretion, and CORT concentrations at the individual level.

### Feather and pCORT concentrations are not correlated

The results of this study clearly indicate that fCORT and pCORT were not correlated in this specific instance. Since nestlings were exposed to ALAN for almost 2 weeks before sampling, we predicted that if ALAN would affect nestling CORT concentrations, it would be easier to detect this effect in the feather compared with the plasma samples, as fCORT represents long-term changes in allostatic load, whereas pCORT represents recent exposure to stressors. While feather CORT can reflect pCORT, correlations may not always be present, especially when elevations in pCORT in response to stressors are modest (Fairhurst et al. 2013; Legagneux et al. 2013). Moreover, the relationship between the two measures is subject to the timing of sample collection relative to stressors and changes in the responsiveness of the HPA axis to such stressors (Fairhurst et al. 2013; Aharon-Rotman et al. 2017). For instance, pCORT values after stress_**‐**_induced stimulation, but not baseline values, correlated with fCORT in red-legged partridges (Bortolotti et al. 2008). Moreover, CORT deposition in feathers may be confounded when feather mass and growth rates are compromised by nutritional stress (Patterson et al. 2015). We did not measure feather growth rate, but anecdotal evidence and clear differences in body size between urban and forest nestlings (Capilla-Lasheras et al. 2017) suggest that nestlings in our urban populations had underdeveloped feathers at the time of sampling, which might have affected fCORT values in unpredictable ways (Jenni-Eiermann et al. 2015).

### Limitations and future directions

The current has limitations that need to be considered to fully comprehend our results and to suggest future directions.

First, it is at present impossible to discern whether the effects of ALAN on CORT of nestlings are direct or indirect. Indirect effects may arise, for instance, via changes in parental care. ALAN has been shown to affect sleep patterns of female great tits during the nestling period (Raap et al. 2015; Sun et al. 2017), which could in turn shift circadian rhythms of their brood. Exposure to ALAN could also affect provisioning rates. Indeed, great tits’ parents feed their nestlings more when exposed to ALAN just outside their nestbox (Titulaer et al. 2012). However, field experiments that have illuminated larger areas of the landscape around nestboxes have found opposite effects, with light pollution decreasing feeding rates of great tits (Welbers et al. 2017). Future studies should attempt to disentangle direct versus indirect routes through which ALAN may affect CORT levels of nestling birds.

Second, the sex of the nestlings used in our experiment was not determined. In one study on great tits, offspring sex ratios did not vary between urban and rural populations (Ágh et al. 2020). However, as glucocorticoid responses might vary between males and females (Goymann 2005; Bonier et al. 2007; Edwards et al. 2013), it will be necessary to also consider sex as an important variable in future studies.

Third, the ALAN treatment might have affected the natural circadian rhythm of CORT, as suggested in previous captive studies (Mishra et al. 2019). While all nestlings in this study were sampled in the morning, exposure to ALAN might have advanced the daily peak of CORT concentration, leading to potential biases in the comparison with dark control nestlings, particularly in the case of plasma samples. Future studies should attempt to obtain multiple samples during the 24 h to fully assess the impact of ALAN on pCORT.

Last, as the blue tit is a cavity nesting-species, the nestlings of this species are not directly exposed to ALAN. This species was used in the current experiment for the ease with which it was possible to manipulate ALAN at the nest, in a highly controlled manner, and because blue tits are a model species in urban ecology. While we recognize that a light intensity of 2 lux is much higher compared with the nocturnal light exposure in natural and nestbox cavities (close to 0 lux), this intensity is within the range of light levels to which birds are exposed to in the urban night outside of nest cavities (Dominoni et al. 2013a). Furthermore, previous data were readily available from the great tit, a closely related species, on their physiological responses to ALAN (de Jong et al. 2017; Raap et al. 2017; Welbers et al. 2017). However, we highlight the need to perform similar experiments in non-cavity-nesting species.

## Conclusions

As wildlife, including birds, are exposed to increasing amounts of anthropogenic stressors as a result of land use change and the destruction of natural habitats, understanding how these processes contribute to HPA development is important (Injaian et al. 2020). The results of this study show that the HPA axis of urban and forest blue tit nestlings responds differently when exposed to ALAN. While it is premature to conclude whether or not the results of this study reflect adaptive response of urban birds to light pollution, the findings suggest that glucocorticoids could be a mechanism by which ALAN affect phenotypic traits of wild birds, and thereby promote physiological adaptation to anthropogenic environments.

## Supplementary Material

icab067_Supplementary_Data

## Data Availability

The data and R code are available from the corresponding author on request.

## Notes

From the symposium “SICB-Wide Symposium: Blinded by the light: Effects of light pollution across diverse natural systems” presented at the virtual annual meeting of the Society for Integrative and Comparative Biology, January 3–7, 2020.
