# Supplementary material for: Feather, But Not Plasma, Glucocorticoid Response to Artificial Light at Night Differs between Urban and Forest Blue Tit Nestlings
Source: Integr Comp Biol. 2021 Jul 17;61(3):1111–21. doi: 10.1093/icb/icab067 (PMC8490687; doi:10.1093/icb/icab067)

Supplementary materials to:

**Feather, but not plasma, glucocorticoid response to artificial light at night differs between urban and forest blue tit nestlings**

Davide M. Dominoni, Dylon Teo, Claire J. Branston, Aryan Jackar, Bedur Faleh A Albalawi, Neil P. Evans

**Supplementary tables**

**Table S1:** Description of study sites.

**Table S2**: Output of final linear mixed effects model testing for the effect of habitat, treatment and their interaction on feather CORT (fCORT) concentrations. fCORT data (+1) was log- transformed to adhere to the assumption of normality of model residuals. Additional explanatory variables were body mass and date of hatching. Random effects included were site and nestbox. Non-significant variables were excluded from the final model.

| **Response: log (fCort + 1)** |  | | |  |  |
| --- | --- | --- | --- | --- | --- |
| *Predictors* | *Estimate* | *SE* | *t* | *p* |  |
| (Intercept) | 1.83 | 0.16 | 11.57 | **<0.001** |  |
| Habitat [forest] | -0.65 | 0.22 | -3.01 | **0.024** |  |
| Treatment [control] | -0.31 | 0.21 | -1.45 | 0.159 |  |
| Habitat * Treatment | 0.62 | 0.3 | 2.11 | **0.044** |  |
|  |  |  |  |  |  |
| *Random Effects* | | | |  |  |
| Residual variance | 0.21 | | |  |  |
| Site variance | 0.004 |  |  |  |  |
| Nestbox variance | 0.12 | | |  |  |
| N _nestboxes/broods_ | 35 | | |  |  |
| N _site_ | 6 |  |  |  |  |
| Observations | 139 | | |  |  |

**Table S3**. Post-hoc tests for model depicted in table S2. Marginal means and confidence intervals (CI) were estimated using the package *emmeans* in R.

| **Post-hoc test fCORT** |  |  |  |  |  |
| --- | --- | --- | --- | --- | --- |
| *Treatment* | *Habitat* | *Estimated mean* | *df* | *lower CI* | *upper CI* |
| ALAN | city | 1.82 | 28.49 | 1.50 | 2.14 |
| control | city | 1.51 | 30.87 | 1.20 | 1.82 |
| ALAN | forest | 1.18 | 26.47 | 0.89 | 1.47 |
| control | forest | 1.49 | 30.11 | 1.19 | 1.79 |

**Table S4**: Output of final linear mixed effects model testing for the effect of habitat, treatment and their interaction on plasma CORT (pCORT) concentrations. pCORT data (+1) was log-transformed to adhere to the assumption of normality of model residuals. Additional explanatory variables were body mass and date of hatching. Random effects included were site and nestbox. Non-significant variables were excluded from the final model.

| **Response: log (pCort + 1)** |  | | |  |
| --- | --- | --- | --- | --- |
| *Predictors* | *Estimates* | *SE* | *t* | *p* |
| (Intercept) | 1.58 | 0.11 | 14.22 | **<0.001** |
| Habitat [forest] | 0.41 | 0.15 | 2.65 | **0.009** |
| Body Mass | -0.23 | 0.08 | -2.93 | **0.004** |
| *Random Effects* | | | |  |
| Residual variance | 0.54 | | |  |
| Nestbox variance | 0 | | |  |
| Site variance | 0 |  |  |  |
| N _nestboxes/broods_ | 34 |  |  |  |
| N _site_ | 6 | | |  |
| Observations | 91 | | |  |

**Table S5**. Post-hoc tests for model depicted in table S4. Marginal means and confidence intervals (CI) were estimated using the package *emmeans* in R.

| **Post-hoc test pCORT** |  |  |  |  |
| --- | --- | --- | --- | --- |
| *Habitat* | *Estimated mean* | *df* | *lower CI* | *upper CI* |
| city | 1.58 | 29.20 | 1.35 | 1.81 |
| forest | 1.99 | 29.68 | 1.77 | 2.21 |

**Table S6**. Output of final linear model testing for the effect of habitat, treatment, fCORT and hatchdate on fledging success. fCORT data (+1) was log-transformed.

| **Response: fledging success (fledglings/hatchlings)** | | | | |  |
| --- | --- | --- | --- | --- | --- |
|  |  |  |  |  |  |
| *Predictors* | *Estimate* | *SE* | *Z* | *p* |  |
| (Intercept) | -19.87 | 9.14 | -2.17 | **0.03** |  |
| Habitat [forest] | 3.08 | 0.87 | 3.59 | **<0.001** |  |
| Treatment [control] | 7.58 | 2.14 | 3.53 | **<0.001** |  |
| log fCORT | 3.2 | 1.02 | 3.13 | **0.002** |  |
| Hatch date | 0.12 | 0.07 | 1.86 | 0.062 |  |
| Treatment * log fCORT | -4.43 | 1.2 | -3.54 | **<0.001** |  |
|  |  |  |  |  |  |
| Observations | 35 | | | |  |

**Table S7**. Table output of final linear model testing for the effect of habitat, treatment, pCORT and hatchdate on fledging success. pCORT data (+1) was log-transformed.

| **Response: fledging success (fledglings/hatchlings)** | | | | |  |
| --- | --- | --- | --- | --- | --- |
|  |  |  |  |  |  |
| *Predictors* | *Estimate* | *SE* | *Z* | *p* |  |
| (Intercept) | 5.1 | 1.22 | 4.16 | **<0.001** |  |
| Habitat [forest] | 1.47 | 0.48 | 3.08 | **0.002** |  |
| log pCORT | -1.5 | 0.53 | -2.8 | **0.005** |  |
| Observations | 33 | | | |  |

**Supplementary figures**

**Figure S1**. Map of study sites. For details on each site (yellow pins, N=6), see Table S1.


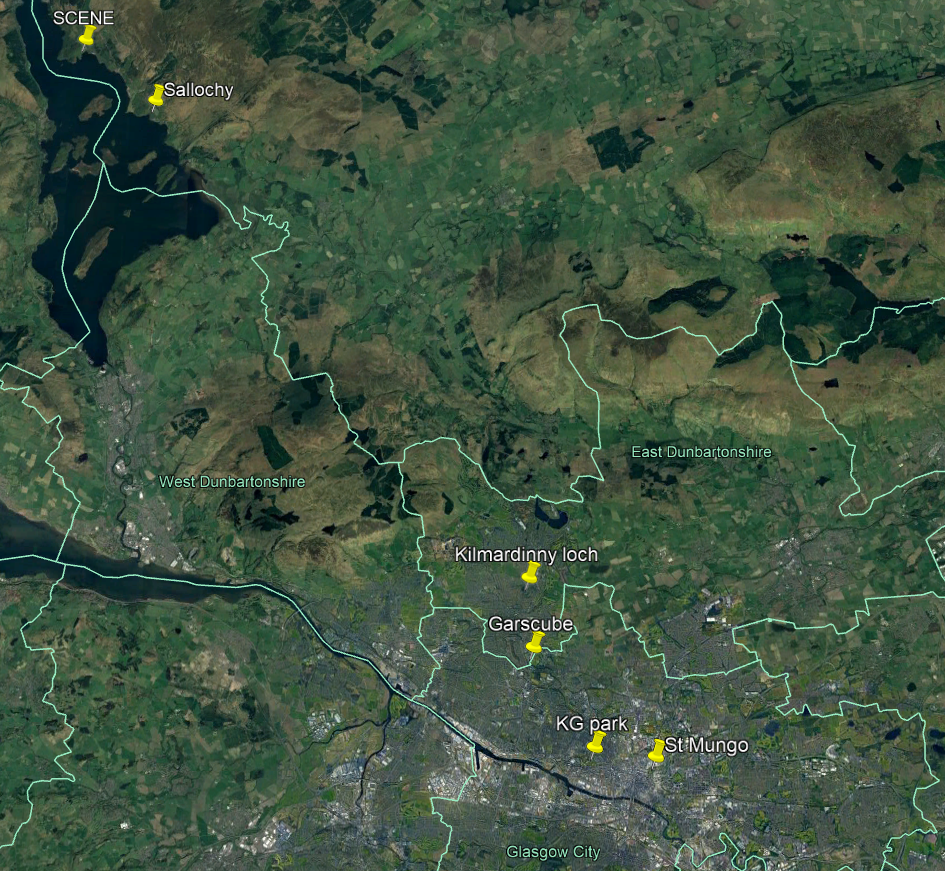


**Figure S2.** Graph depicting the relationship between feather mass and feather corticosterone concentration. This relationship was not significant (ANOVA, F_1,137_ = 0.01, p = 0.91).


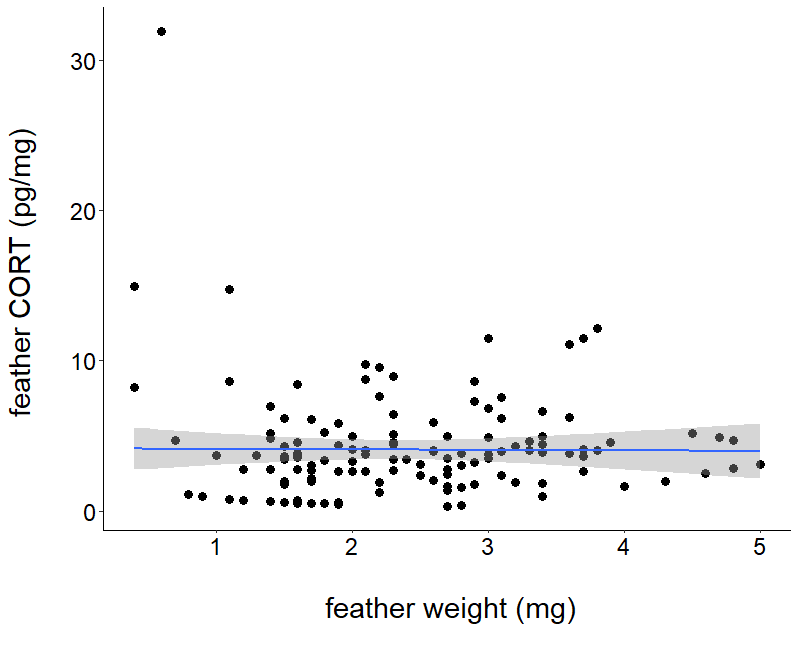


**Figure S3**. Parallelism check for the corticosterone assay showing that the dilution series of the feather extract (open symbols, dashed line) lies parallel with the standard (filled symbols solid line).

**
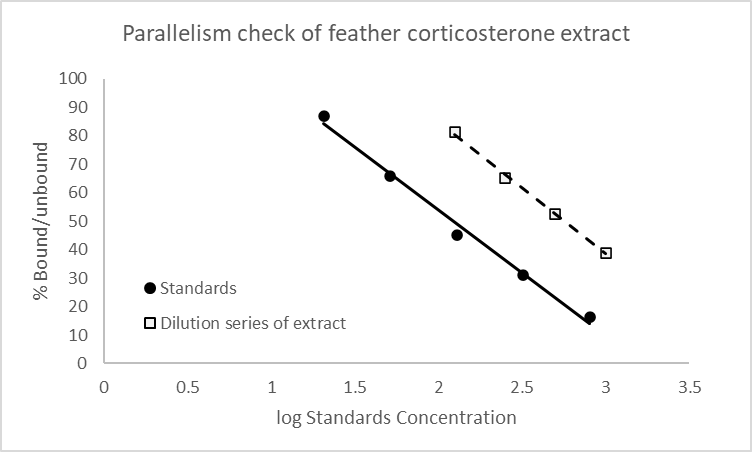
**

**Figure S4**. Scatterplot depicting the lack of correlation between pCORT and fCORT.


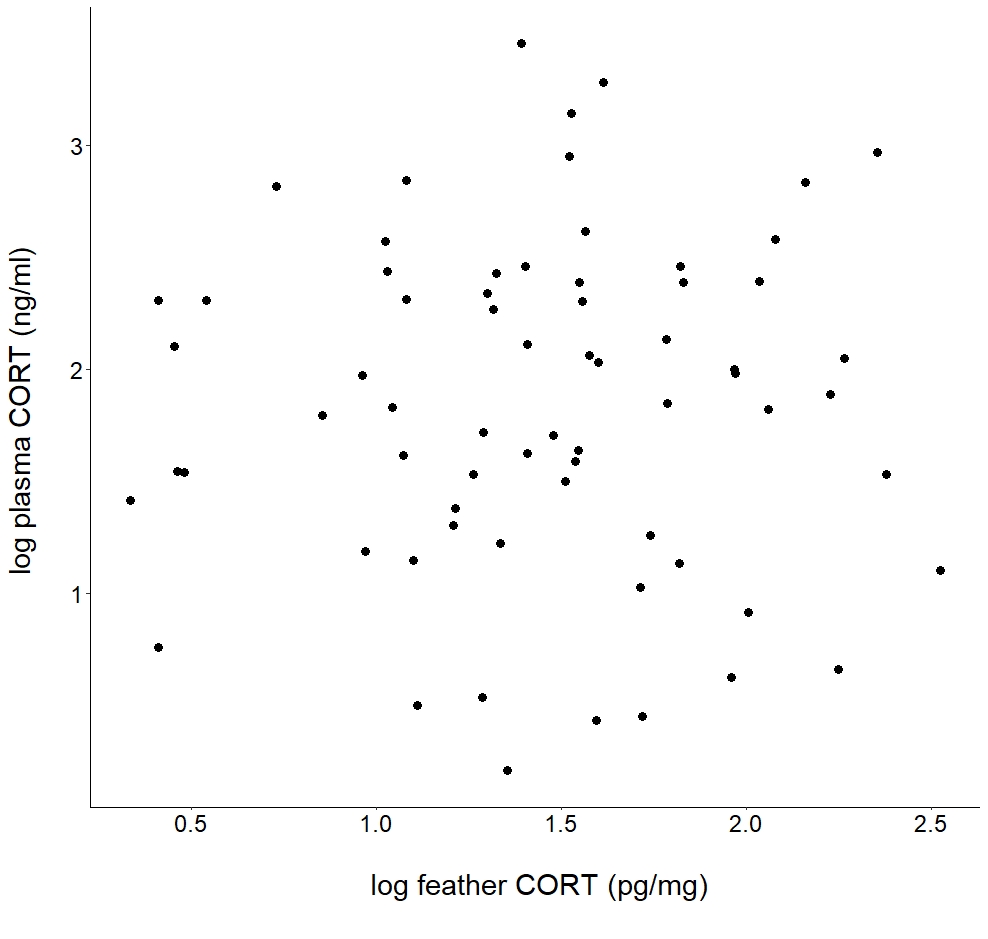

Supplement: icab067_Supplementary_Data [file icab067_supplementary_data.docx]
